# Primary leiomyosarcoma of the spine: an analysis of imaging manifestations and clinicopathological findings

**DOI:** 10.1186/s13244-022-01336-y

**Published:** 2022-12-15

**Authors:** Jiahui Zhang, Yongye Chen, Xiaoying Xing, Qizheng Wang, Ke Liu, Enlong Zhang, Ning Lang

**Affiliations:** 1 Department of Radiology, Peking University Third Hospital, 49 North Garden Road, Haidian District, Beijing, 100191 People’s Republic of China; 2 Department of Radiology, Peking University International Hospital, Beijing, People’s Republic of China

**Keywords:** Spine, Magnetic resonance imaging, Computed tomography

## Abstract

**Background:**

Primary leiomyosarcoma of the spine is extremely rare and lacks specific clinical symptoms. This study investigated the imaging manifestations and clinicopathological findings of primary leiomyosarcoma of the spine, aiming to improve the radiologists’ understanding of the disease and reduce misdiagnoses.

**Methods:**

The clinical, imaging, and pathological manifestations in eleven patients with pathologically confirmed primary leiomyosarcoma of the spine were retrospectively analyzed. The imaging features analyzed included lesion location, shape, border, size, and density/intensity, and adjacent bone destruction status, residual bone trabeculae, vertebral compression, and contrast enhancement.

**Results:**

The patients’ primary clinical symptom was usually focal pain. Primary leiomyosarcoma of the spine was mostly a solitary lesion and tended to occur in the posterior elements. The tumors had a lobulated shape with osteolytic bone destruction, ill-defined borders, and could involve multiple segments. Computed tomography (CT) examination showed isodense masses. Six patients showed residual bone trabeculae. Two patients had miscellany T2-weighted imaging (T2WI) signals, while the tumor and spinal cord of the remaining patients were isointense on T1-weighted imaging (T1WI) and T2WI. Among the seven patients who underwent contrast-enhanced scanning, six displayed homogeneous enhancement. Eight patients underwent gross-total tumor resection with no recurrence.

**Conclusions:**

Primary leiomyosarcoma of the spine tends to be a solitary lesion in the posterior elements and appears as a lobulated mass with osteolytic bone destruction and an ill-defined border. The tumor and spinal cord can be isointense on T1WI and T2WI. Contrast-enhanced scanning displays homogeneous enhancement. The lesion tends not to recur after surgical gross-total tumor resection.

## Key points

1. Primary leiomyosarcoma of the spine is extremely rare.
2. Some imaging features could be valuable for diagnosing the disease.
3. The treatment of choice is surgical gross-total resection of the tumor.

## Background

Leiomyosarcoma is a rare malignant spindle cell neoplasm that develops in smooth muscles and accounts for nearly 7% of the soft tissue sarcomas [1, 2]. It usually occurs in the retroperitoneum and peritoneal cavity, in organs such as the gastrointestinal tract and the uterus. Leiomyosarcoma in the bones is rare, accounting for 0.06% of the primary and 0.14% of the malignant bone tumors [3]. Primary leiomyosarcomas in the spine are extremely rare, with only a dozen cases reported in the literature [4]. To the best of our knowledge, no series of imaging manifestations of primary leiomyosarcoma of the spine is available in the literature. This study reviewed the clinical features, imaging manifestations, and pathological findings of eleven cases of pathologically confirmed primary leiomyosarcoma of the spine, diagnosed over the past sixteen years, to improve the understanding of the disease and reduce misdiagnoses.

## Materials and methods

### Patients

The Institutional Review Board of our hospital approved this retrospective study, and the requirement for informed consent was waived. This study retrospectively reviewed the clinical features, imaging manifestations, and pathological findings of eleven cases of pathologically confirmed primary leiomyosarcoma of the spines between January 2006 and May 2022. Of the eleven patients, five were male, and six were female. All patients underwent computed tomography (CT) examination, which included contrast-enhanced CT examination in three. Ten patients underwent magnetic resonance imaging (MRI), which included contrast-enhanced MRI examination in six. All lesions were surgically resected or subjected to CT-guided biopsy, and the diagnosis was confirmed by pathology.

### Imaging examination

CT examination was made using a Siemens Somatom Definition Flash dual-source scanner (Siemens, Erlangen, Germany) or GE Discovery CT 64 VCT (GE Medical System, Chalfont St. Giles, UK). The parameters were: tube potential, 120 kV; tube current, 163–300 mAs; thread pitch, 0.980; slice thickness, 3 mm; slice spacing, 3 mm. The sagittal and coronal views were reconstructed. For enhanced scanning, a non-ionic contrast agent (Iopamiro; 350 mg I/mL) was injected via the elbow vein, using a high-pressure injection system at a dose of 2 mL/kg and a rate of 3 mL/s.

MRI scanning was performed using a GE Discovery MR750 3.0 T scanner (GE Healthcare, Piscataway, NJ, USA) or a Siemens Magnetom Trio Tim 3.0 T scanner (Siemens, Erlangen, Germany). Standard T1-weighted imaging (T1WI) and T2-weighted imaging (T2WI) scans were acquired with a surface coil, slice thickness of 3.0 mm, and slice spacing of 3.3 mm. The sequence scanning parameters were: T1WI: repetition time (TR), 400–800 ms and echo time (TE), 10–30 ms; T2WI: TR, 2500–4000 ms and TE, 50–120 ms. Scanning sequences included T1WI and axial, sagittal, coronal, and fat-suppressed T2WI. Gadolinium (Gadopentetate dimeglumine; Beilu Pharmaceutical, Beijing, China) was used as the contrast enhancement agent at 0.2 mL/kg, injected via an elbow vein under high pressure at a rate of 1.0 mL/s. Fat-suppressed T1WI was performed after injection with the following parameters: TR, 571–652 ms; TE, 9.8–11.2 ms.

### Image analysis

Two radiologists experienced in spinal tumor diagnosis (X.Y.X. with ten years of experience and J.H.Z. with five years of experience) independently analyzed the CT and MRI images. Details noted included the lesion location, shape, border, mass size, and density/intensity, and the adjacent bone destruction status, vertebral compression, nerve root compression, spinal cord compression, and degree of contrast enhancement. Inconsistencies were resolved by discussion and consultation.

### Pathological examination

All lesions were surgically resected or subjected to CT-guided biopsy. For CT-guided biopsy, the patients were placed in a lateral or prone position, the region was sterilized and anesthetized layer-by-layer with 1% lidocaine. A coaxial biopsy needle was used as the trocar. An automatic biopsy needle was used to obtain the specimens, which were fixed in 10% formalin. Following biopsy, the patients were rescanned to rule out any complications. Pathological examinations included routine hematoxylin and eosin (HE) and immunohistochemical staining. The markers used included smooth muscle actin (SMA), vimentin, epithelial membrane antigen (EMA), S-100, and CD34.

## Results

### Clinical features

The median patient age was 55 (range 17–77) years. One patient had multiple lesions. The lesion locations of the remaining ten patients were as follows: cervical vertebrae in three; thoracic vertebrae in five; lumbar vertebrae in one; thoracolumbar region in one. The main clinical manifestations were pain, weakness, numbness, and movement restriction. Among the eleven patients, one underwent radiotherapy, two underwent subtotal resection, and the remaining eight underwent gross-total tumor resection with postoperative radiotherapy in five. Re-examinations were performed 3, 6, and 12 months post-operatively. Follow-up was performed once every 6–12 months if no recurrence was detected. The two patients who underwent subtotal resection showed disease progression at the 6-month follow-up examination. The patient who underwent radiotherapy showed no progression at the 3-month follow-up assessment. The eight patients treated by gross-total tumor resection had no recurrence. Non-recurrent patients were those with no tumor detected for at least one year. The patient information is presented in Table 1.

**Table 1:** General information of the 11 patients with primary leiomyosarcoma of the spine

No	Gender	Age (years)	Symptoms and medical history	Lesion segment	Treatment methods	Follow-up time (months)	Prognosis
1	Female	55	Waist pain for six months, weakness in both lower limbs for one month	L3	Gross-total resection and radiotherapy	83	Recurrence-free survival
2	Male	54	Left waist and abdomen pain and movement restriction for over three months	T11	Gross-total resection and radiotherapy	56	Recurrence-free survival
3	Male	67	Waist pain and movement restriction for six months	T10	Subtotal resection	6	Progression
4	Female	67	Neck pain for two months, movement restriction for 15 days	C2-C3	Subtotal resection	6	Progression
5	Female	17	Left chest and back pain for four months	T10-L1	Gross-total resection	51	Recurrence-free survival
6	Female	73	Left shoulder and back numbness for over three months	C4	Gross-total resection	38	Recurrence-free survival
7	Female	44	Waist pain for two years, right lower limb numbness for over three months	T11-T12	Gross-total resection	20	Recurrence-free survival
8	Male	30	Back pain for four months	T7-T8	Gross-total resection and radiotherapy	11	Recurrence-free survival
9	Female	57	Left waist and back pain for one year	T11	Gross-total resection and radiotherapy	10	Recurrence-free survival
10	Male	37	Neck pain for one year	C2	Gross-total resection and radiotherapy	5	Recurrence-free survival
11	Male	77	Waist and back pain and movement restriction for three months	Multiple	Radiotherapy	3	Progression-free survival

### Imaging manifestations

**Fig. 1:**
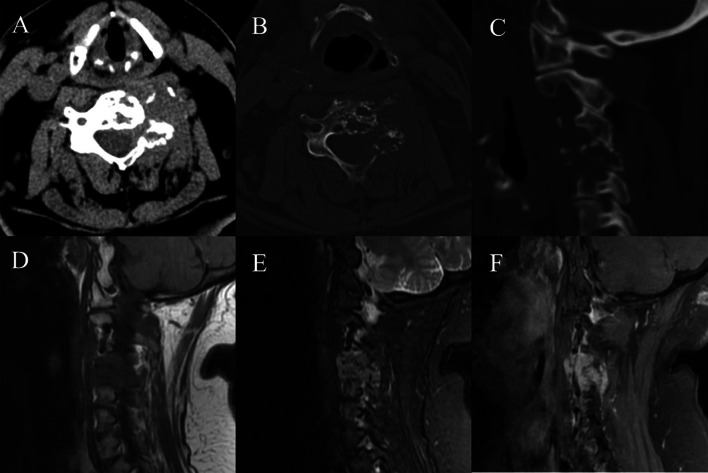
A 73-year-old female with C4 primary leiomyosarcoma. **A** Axial computed tomography (CT) showed an ill-defined isodense mass in the vertebral body and posterior elements of C4; **B** axial CT bone window showed osteolytic bone destruction, with residual bone trabeculae; **C** sagittal CT bone window showed osteolytic bone destruction; **D** T1 weighted imaging (T1WI) in sagittal view showed an isointense mass; **E** T2 weighted imaging (T2WI) in sagittal view showed an isointense mass; **F** Magnetic resonance imaging (MRI) enhanced scanning showed homogeneous enhancement of the mass that appeared lobulated

**Fig. 2:**
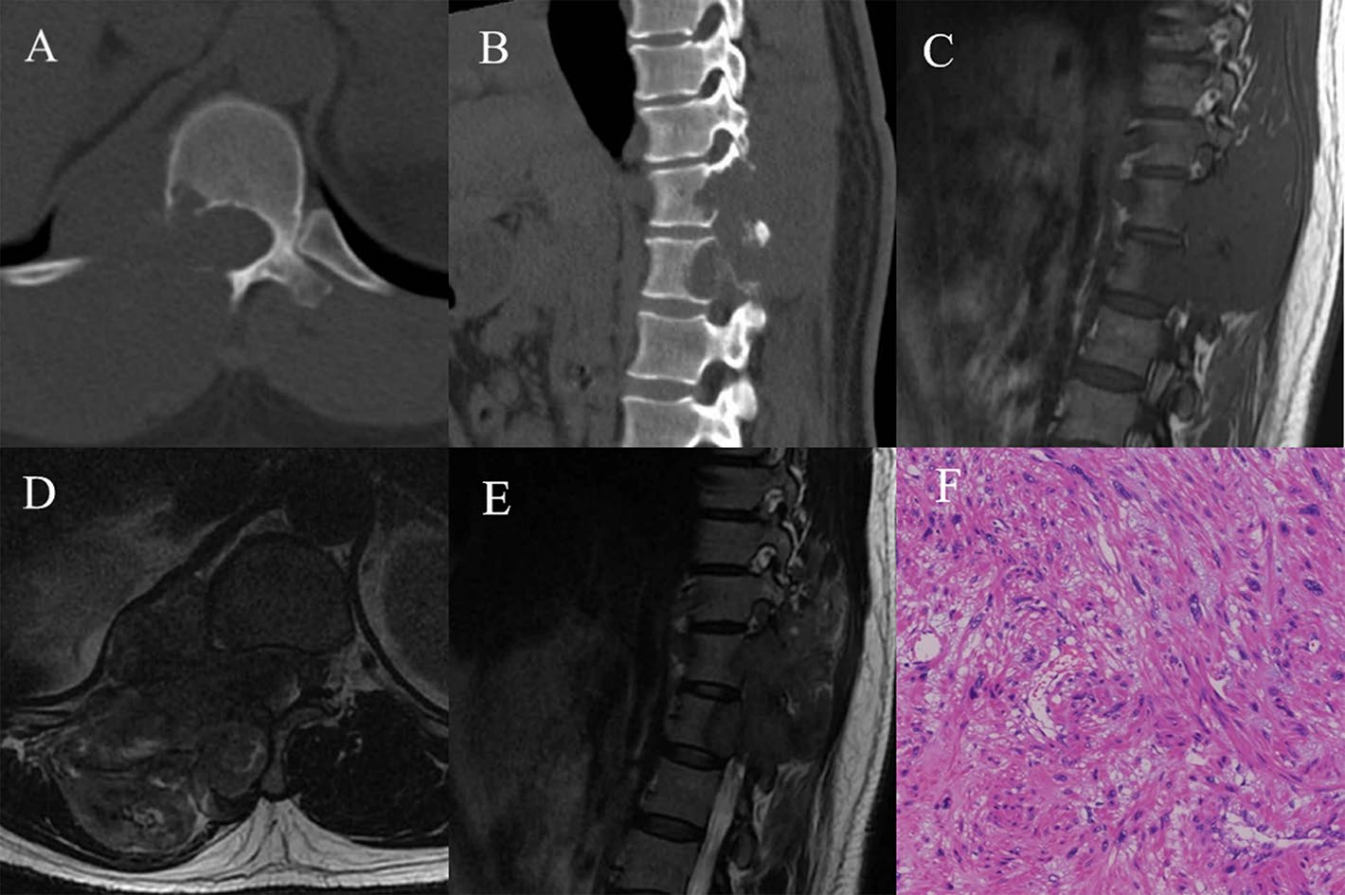
A 44-year-old female with T11–T12 primary leiomyosarcoma. **A** Axial computed tomography (CT) bone window showed that the mass destroyed the surrounding bone; **B** Sagittal CT bone window showed bone destruction of T11–T12 vertebral body and posterior elements, with residual bone trabeculae; **C** T1 weighted imaging (T1WI) in sagittal view showed an isointense mass; **D** T2 weighted imaging (T2WI) in axial view showed a lobulated mass with ill-defined border; **E** T2WI in sagittal view showed an isointense mass; **F** The interstitial tumor showed diffuse spindle cell hyperplasia with many nuclear fissions (HE; ×400)

**Fig. 3:**
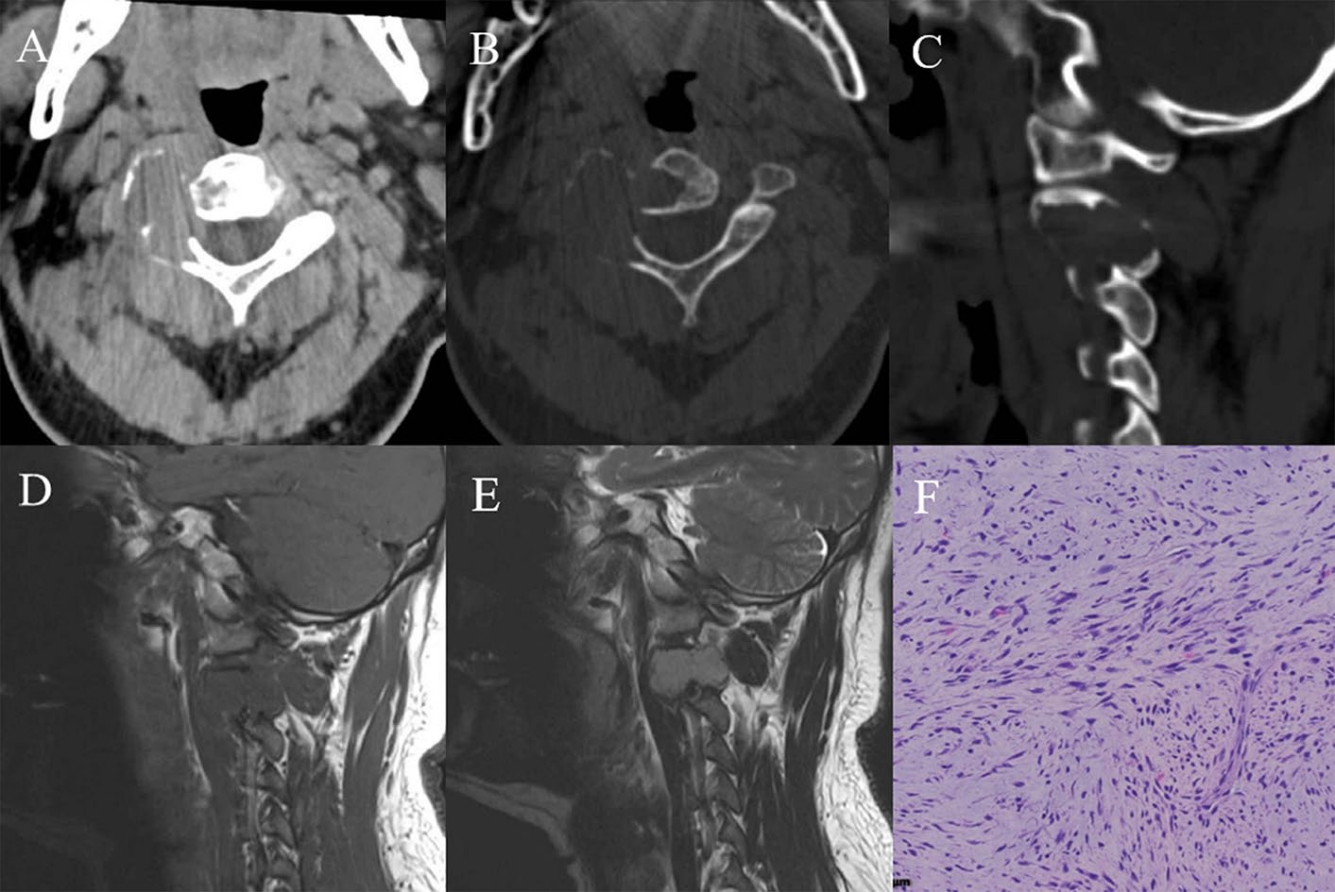
A 37-year-old male with C2 primary leiomyosarcoma. **A** Axial computed tomography (CT) showed an ill-defined isodense mass in the vertebral body and posterior elements of C2; **B** Axial CT bone window showed expansive bone destruction; **C** Sagittal CT bone window showed bone destruction in the vertebral body and posterior elements of C2; **D** T1 weighted imaging (T1WI) in sagittal view showed an isointense mass; **E** T2 weighted imaging (T2WI) in sagittal view showed an isointense mass; **F** The spindle cell in the tumor differentiated into densely packed smooth muscle cells; no tumor necrosis was observed (HE, ×400)

**Fig. 4:**
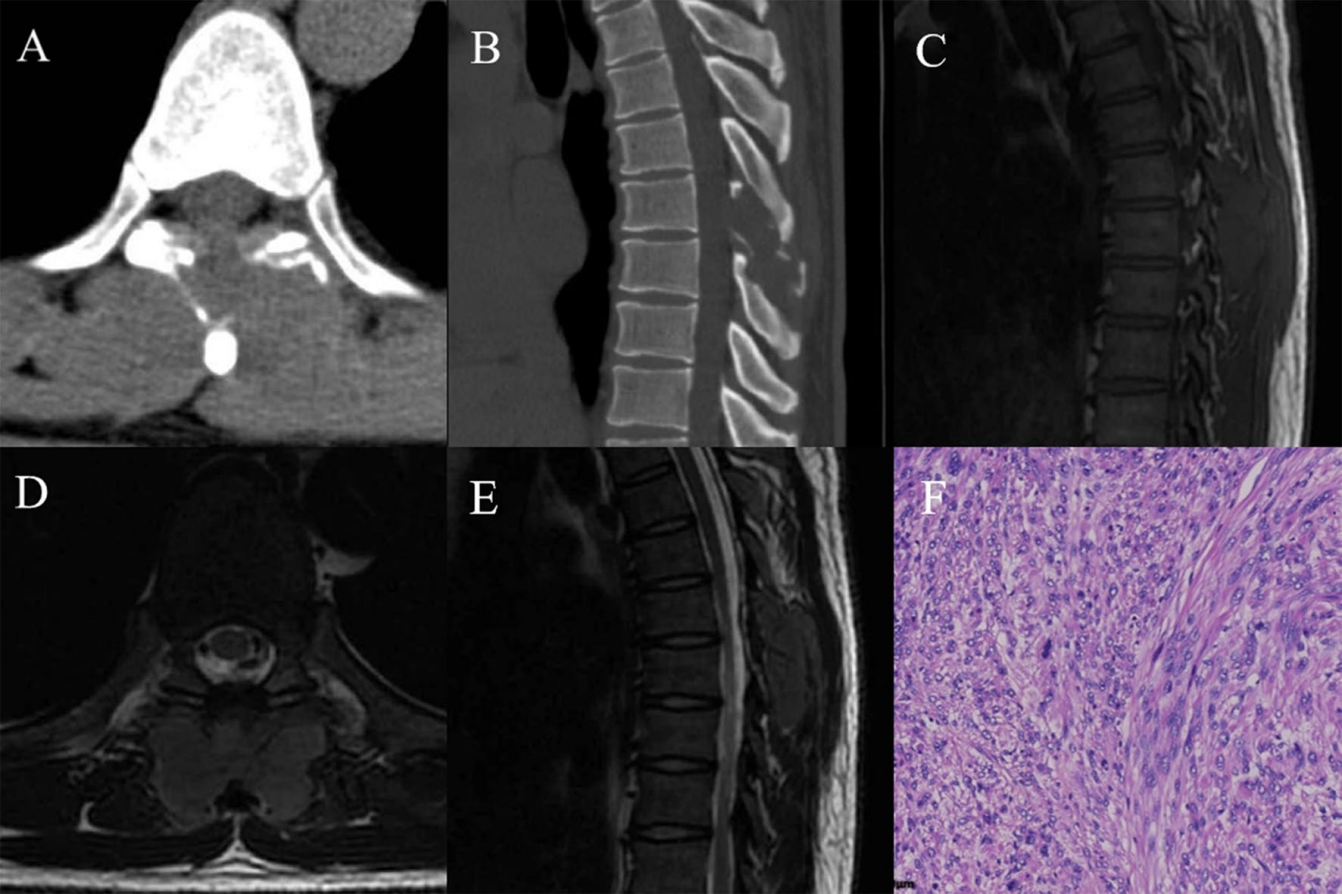
A 30-year-old male with T7–T8 primary leiomyosarcoma. **A** Axial computed tomography (CT) showed an ill-defined isodense mass in the posterior elements of T7–T8; **B** Sagittal CT bone window showed bone destruction in the posterior elements of T7–T8; **C** T1 weighted imaging (T1WI) in sagittal view showed an isointense mass; **D** T2 weighted imaging (T2WI) in axial view showed a lobulated mass; **E** T2WI in sagittal view showed an isointense mass; **F** the diffuse spindle cell hyperplasia confirmed to be a feature of interstitial tumors. The tumor cells were visibly atypical, and the nuclear fission rate was high (HE, ×400)

The largest single-lesion tumor was in the T10-L1 segment. Vertebral body and/or posterior element involvement were limited to a single level in six patients and showed multi-level involvement in five. The lesions were centered in the posterior elements and extended into the vertebral body in eight patients, while the lesions were centered in the vertebral body and extended into the posterior elements in the remaining three. Masses that invaded the vertebrae or paravertebral tissues in nine patients displayed a lobulated shape. Osteolytic bone destruction was noted in eight patients, and expansive bone destruction in three. Nine of the eleven patients showed ill-defined borders, while the border of the remaining two was well defined. Six patients had residual bone trabeculae. The vertebrae of three lesions showed pathological compression fractures. All lesions had the same density as the surrounding muscles, with a mean radiodensity of 50 Hounsfield units (HU). MRI was isointense to the spinal cord signal in T1WI of ten patients and T2WI of eight. Two patients showed miscellany intensity on T2WI. Spinal cord compression was noted in four patients, and nerve root compression in seven. Among the seven patients who underwent contrast-enhanced scanning, six exhibited homogeneous enhancement. The details of the imaging manifestations are shown in Table 2 and Figs. 1, 2, 3 and 4.

**Table 2:** Imaging manifestations of the 11 patients with primary leiomyosarcoma of the spine

No	Location	Shape	Border	Bone destruction	Residual bone trabeculae	Compression fractures	CT value (HU)	T1WI	T2WI	Enhanced features	Spinal cord compression	Nerve root compression	Mass size (cm)
1	L3 vertebral body and posterior elements	Lobulated	Ill-defined	Osteolytic	No	Yes	53	Isointense	Isointense	Obviously homogeneous	Yes	Yes	5.5 × 4.2 × 2.0
2	T11 vertebral body and posterior elements	Lobulated	Ill-defined	Osteolytic	Yes	Yes	43	Isointense	Isointense	Obviously homogeneous	Yes	Yes	6.5 × 5.1 × 1.8
3	T10 vertebral body	Lobulated	Well-defined	Osteolytic	Yes	No	61	Isointense	Miscellany intensity	Obviously heterogeneous	Yes	No	3.4 × 3.4 × 2.4
4	C2-C3 vertebral body	Irregular	Ill-defined	Osteolytic	No	No	54	–	–	Obviously homogeneous	No	Yes	2.3 × 2.0 × 1.4
5	T10-L1 vertebral body and posterior elements	Lobulated	Well-defined	Osteolytic	No	No	50	Isointense	Miscellany intensity	–	No	Yes	4.1 × 5.9 × 11.0
6	C4 vertebral body and posterior elements	Lobulated	Ill-defined	Osteolytic	Yes	No	58	Isointense	Isointense	obviously homogeneous	No	Yes	3.4 × 3.5 × 2.4
7	T11-T12 vertebral body and posterior elements	Lobulated	Ill-defined	Expansive	Yes	No	40	Isointense	Isointense	–	Yes	Yes	7.3 × 6.5 × 9.6
8	T7-T8 posterior elements	Lobulated	Ill-defined	Expansive	Yes	No	39	Isointense	Isointense	–	No	No	3.6 × 3.2 × 5.7
9	T11 posterior elements	Lobulated	Ill-defined	Osteolytic	No	No	37	Isointense	Isointense	obviously homogeneous	No	No	2.5 × 1.0 × 5.1
10	C2 vertebral body and posterior elements	Lobulated	Ill-defined	Expansive	Yes	No	59	Isointense	Isointense	obviously homogeneous	No	Yes	2.7 × 3.3 × 2.3
11	Multiple vertebral bodies	Irregular	Ill-defined	Osteolytic	No	Yes	60	Isointense	Isointense	–	No	No	–

The patient number in Table 2 is consistent with that in Table 1

### Pathological findings

Three patients underwent CT-guided core needle biopsy, two underwent subtotal resection, and eight underwent gross-total tumor resection. The specimen pathological result showed diffuse spindle cell hyperplasia, a characteristic of mesenchymal tumors, with cellular atypia, rich cytoplasm, and widespread nuclear fission. Immunohistochemical staining outcomes were as follows: SMA (+), vimentin (+), EMA (−), S-100 (minor +), CD34 (−).

## Discussion

Leiomyosarcoma, first reported in 1965, is a rare sarcoma type, accounting for < 0.7% of all primary malignant bone tumors [5]. The etiology of primary leiomyosarcoma of the bone is unclear. The histological features of leiomyosarcoma of the bone are the same as those of leiomyosarcoma found in other tissues. Some studies suggested that the lesions might be derived from smooth muscle cells within the bone marrow cavity or mesenchymal stem cell that have not differentiated into smooth muscle cells [6–8]. Primary leiomyosarcoma rarely occurs in the bones. When it does, the epiphysis of the long bones in the lower limb, particularly near the knee joint, is the most common location [8]. Leiomyosarcoma in the spine is extremely rare, with the thoracic vertebrae being the most affected segment [4, 9]. Leiomyosarcoma can occur at any age, but it is more common in middle-aged individuals. The incidence rates in males and females are roughly equal. Primary leiomyosarcoma of the spine lacks specific clinical symptoms. Patients usually present with focal pain that might be accompanied by weakness, numbness, movement restriction, or paresthesia of the extremities [2]. These manifestations depend primarily on the location and size of the lesion. Primary bone leiomyosarcoma might form soft tissue masses and cause pathological fractures [8]. When the tumor encroaches and presses the adjacent nerves or spinal cord, it could cause movement and sensory disturbances in the corresponding innervation area [10]. The patients in our study presented with typical symptoms such as focal pain, weakness, numbness, and movement restriction, with three patients presenting with pathological compression fractures, consistent with previous reports [4, 8].

Standard radiography has limited diagnostic value, and imaging manifestations often lack specificity. CT and MRI examinations can clearly show changes in the bone and invasion of the surrounding soft tissue. CT is more valuable in detecting changes in the bone structure such as bone destruction and residual bone trabeculae than MRI. Previous studies have suggested that primary bone leiomyosarcoma lacks specific imaging manifestations [7, 8, 11]. We found in this study that some imaging features appeared frequently, providing some valuable information for diagnosis of primary leiomyosarcoma of the spine. Primary leiomyosarcoma of the spine usually presents as s solitary lesion, tends to occur in the posterior elements, and often has a lobulated shape with osteolytic bone destruction and ill-defined borders. Furthermore, a single lesion might involve multiple spinal segments. CT examination of our patients demonstrated that some had residual bone trabeculae and pathologic compression fractures. T1WI and T2WI scans of eight patients were isointense to the spinal cord, and contrast-enhanced scans displayed apparent homogeneous enhancement. We speculate that these findings are related to the dense arrangement and large nuclear-cytoplasmic ratio of the tumor cells.

The incidence of primary leiomyosarcoma of the spine is extremely low. In the absence of specific clinical symptoms, conclusive diagnosis needs pathological findings. Leiomyosarcoma pathology is divided into normal, epithelial-like, and polymorphic types [12]. The histopathological features of primary leiomyosarcoma of the bone depending on the histological grade [10]. Well-differentiated tumors consist of a few spindle-shaped cells arranged in bundles, with thin and granular cytoplasm and relatively dense fibrous components. The nuclei are round and blunt at both ends, with mild nuclear atypia and few independent intact cells. High-grade leiomyosarcoma cells are densely arranged, independent intact cells are common, diverse-sized and disordered spindle cells, and prominent nuclear atypia [10]. Microscopic examination shows elongated and spindle-shaped tumor cells, rich in cytoplasm, with many nuclear fissions. We have also observed giant cells with obscure necrosis. Routine HE staining often cannot distinguish between normal tissue and undifferentiated sarcoma, so immunohistochemical staining plays a very important role in diagnosing primary leiomyosarcoma of the spine. Immunohistochemical staining of spinal leiomyosarcomas showed positive expression of tumor cell SMA and vimentin, negative expression of CD34 and EMA, and focal weakly positive expression of the S-100 protein [13]. SMA is the most sensitive and relatively specific marker of bone leiomyosarcoma [14]. All eleven cases in this study showed pathological features typical to bone leiomyosarcoma.

Primary leiomyosarcoma of the spine must be distinguished from other spine tumors. First, bone metastasis of other leiomyosarcomas such as those of the gastrointestinal tract, uterus, kidney, and other soft tissues should be ruled out [2, 9]. This possibility can be ruled out by checking the patient's cancer history and performing conventional imaging examinations [4]. Second, primary leiomyosarcoma of the spine should be differentiated from plasmacytoma that often occurs in older adults. Both can be isointense to the spinal cord in T2WI with apparent homogeneous enhancement. However, beyond the background osteoporosis, bones with plasmacytomas are more prone to compression fracture, and the shape of soft tissue mass is irregular or almost round. Primary leiomyosarcoma of the spine should also be distinguished from giant-cell tumor of the bone (GCTB) as both have similar clinical symptoms. However, spinal GCTB often show miscellany intensity on MRI, and mostly affect the vertebral body rather than the posterior elements [15, 16].

Primary leiomyosarcoma of the spine is a highly malignant tumor. Gross-total resection with microscopically negative margins is the leading treatment method [2, 14, 17], aiming to reduce postoperative recurrence rate. It is sometimes combined with contemporaneous or staging-dependent radical resection to relieve nerve compression and pain. Pedicle screw or anterior fixation is used to restore spinal stability. Radiotherapy and chemotherapy can help relieve the clinical symptoms. However, some scholars believe that primary leiomyosarcoma of bones is insensitive to these treatments, which cannot improve the surgical outcomes significantly [13, 14, 17]. Therefore, the effect of postoperative radiotherapy and chemotherapy on prolonging the patients’ survival remains controversial. In our study, the eight patients who underwent gross-total tumor resection showed no recurrence, suggesting that primary leiomyosarcoma of the spine was not easy to recur after complete resection. Conversely, the two patients who underwent subtotal resection showed disease progression by the 6-month follow-up. One patient underwent radiotherapy alone; however, the effect of radiotherapy remains to be proven as the follow-up time in this case was short.

This study represents a preliminary summary of the imaging manifestations and clinicopathological findings of primary leiomyosarcoma of the spine. Although some valuable findings were obtained, the study had several limitations. First, the study was on very rare disease, vastly limiting the number of cases we could assess. Second, the data integrity cannot be guaranteed since the imaging examinations of some patients were not comprehensive. In the future, we will expand the sample to validate and refine our results.

## Conclusion

Primary leiomyosarcoma of the spine is extremely rare and lacks specific clinical symptoms. The imaging manifestations had certain specificity. These tumors were solitary lesions that tended to occur in the posterior elements and appear as lobulated masses with osteolytic bone destruction and ill-defined borders. Furthermore, single-lesion primary leiomyosarcoma of the spine can involve multiple segments. These tumors can be isointense to the spinal cord on T1WI and T2WI. Contrast-enhanced scanning displayed clear homogeneous enhancement. Radiologists should consider primary leiomyosarcoma of the spine when observing these imaging features. The final diagnosis requires confirmation by pathology and immunohistochemical staining. Recurrence rarely occurs after gross-total tumor resection.

## Data Availability

The datasets used and/or analyzed during the current study are available from the corresponding author on reasonable request.

## Abbreviations

CT: Computed tomography
EMA: Epithelial membrane antigen
GCTB: Giant cell tumor of bone
HE: Hematoxylin and eosin
HU: Hounsfield units
MRI: Magnetic resonance imaging
SMA: Smooth muscle actin
T1WI: T1-weighted imaging
T2WI: T2-weighted imaging
TE: Echo time
TR: Repetition time
